# Anxiety, depression, and worries in advanced Parkinson disease during COVID-19 pandemic

**DOI:** 10.1007/s10072-021-05286-z

**Published:** 2021-05-04

**Authors:** Elisa Montanaro, Carlo Alberto Artusi, Cristina Rosano, Carlotta Boschetto, Gabriele Imbalzano, Alberto Romagnolo, Marco Bozzali, Mario Giorgio Rizzone, Maurizio Zibetti, Leonardo Lopiano

**Affiliations:** 1 Neurology 2 Unit, A.O.U. Città della Salute e della Scienza di Torino, Corso Bramante 88, 10126 Torino, Italy; 2 Department of Neuroscience “Rita Levi Montalcini”, University of Torino, Via Cherasco 15, 10126 Torino, Italy; 3 Department of Neuroscience, Brighton and Sussex Medical School, Brighton, UK

**Keywords:** Parkinson disease, COVID-19, Anxiety, Worries, Depression

## Abstract

**Background:**

The psychological impact of the COVID-19 outbreak and lockdown on frail populations with advanced Parkinson disease (APD) and their caregivers may present with peculiar features and require specific interventions.

**Methods:**

We enrolled here 100 APD patients and 60 caregivers. Seventy-four patients were treated with device-aided therapies (DAT) and 26 with standard medical treatment (SMT). Through a telephonic interview, subjects underwent the Hospital Anxiety and Depression Scale (HADS-A; HADS-D), and an ad hoc questionnaire to explore thoughts and emotions related to the pandemic.

**Results:**

Depression was observed in 35% of APD patients and anxiety in 39%, with a significant reduction of the latter after the lockdown (*p*= 0.023). We found a significant correlation between the type of therapy and the HADS-A score (*p*= 0.004). Patients’ main worries were as follows: a possible higher risk of COVID-19 infection (25%), interruption of non-pharmacological treatments (35%), interruption of outpatient clinics (38%), PD complications related to COVID-19 (47%). Patients treated with DAT manifested worries about device-related issues and risk for caregivers’ infection. The 40% of caregivers showed anxiety, while the 21.7% of them showed depression.

**Conclusion:**

Our study reveals a higher prevalence of anxiety and the presence of peculiar worries and needs in APD patients during the pandemic alongside psychological sequelae of their caregivers. These findings are important for neurologists and healthcare services to foster strategies for the management of psychological distress in both patients and caregivers.

**Supplementary Information:**

The online version contains supplementary material available at 10.1007/s10072-021-05286-z.

## Introduction

On 11 March 2020, the World Health Organization declared COVID-19 a global pandemic [1]. To prevent contagion, many countries adopted extraordinary measures such as a lockdown of social and working activities, with significant psychological effects on the population [2]. Particular attention should be paid to the impact of COVID-19 pandemic on more vulnerable individuals who suffer from chronic diseases, such as people with Parkinson disease (PD). Due to their pathological condition, PD patients are indeed at higher risk for developing neuropsychiatric symptoms [3].

During pandemic, an increase of psychological distress, depression, and anxiety have been observed in PD patients [4–6], which are at least partially related to individual perception of higher risk for a worse infection outcome [7] and reduced access to healthcare services [8].

Importantly, higher distress was observed also in caregivers of patients with PD [4]. In such a complex picture, a more specific characterization of the psychological impact of COVID-19 on PD patients and their families is needed, with a special focus on the interaction with the heterogeneous motor and nonmotor symptoms occurring at different clinical stages. Advanced PD (APD) is characterized by an increased frailty related to symptoms’ severity and motor complications that require frequent therapeutic adjustments [9]. Moreover, APD patients may be treated by device-aided therapies (DAT), requesting regular follow-up visits in highly specialized clinical settings [10].

Here, we investigated the psychological impact of the COVID-19 outbreak on APD patients and their caregivers by assessing distress, worries, depressive, and anxious symptoms; in addition, we evaluated potential differences between patients treated with DAT compared to those on standard medical treatment (SMT). We hypothesized an increasing impact of COVID-19 outbreak on PD patients and their caregivers according to disease severity and treatment complexity.

## Methods

### Study population

Using the electronic database of the Movement Disorder Center of the University Hospital of Turin (Italy), we included all non-demented consenting patients treated with DAT at our center in the last 5 years, a group of randomly selected non-demented APD patients treated with SMT (recruited among a group of consecutive, consenting patients who attended our center before the lockdown), and their caregivers (from 59% of patients treated with deep brain stimulation (DBS), 65% of patients treated with levodopa/carbidopa intestinal gel infusion (LCIG), and 58% of patients on SMT). APD was defined as persistence of motor fluctuations and/or troublesome dyskinesia limiting the activities of daily living in spite of repeated adjustments of medication [9].

Patients and caregivers enrolled in the current study were evaluated by phone interview from April 2020 to May 2020 (T0), which was the lockdown interval decided by the Italian Government. Patients were re-evaluated after lockdown conclusion from June 2020 to August 2020 (T1). The Local Ethical Committee approved the study protocol (Protocol number: 00194/2020) and each participant gave verbal informed consent.

### Data collection

All participants were evaluated with the Hospital Anxiety and Depression Scale (HADS) at T0 and T1 to obtain a validated and formal quantification of their depressive and anxious symptoms [11] considering values ≥ 8 as cut-off. We also administered a questionnaire specifically developed to investigate thoughts, fears, and emotions related to COVID-19 outbreak and lockdown, consisting of 22 questions (Supplementary Material 1) and divided into 3 sections:

- Section 1: distress and worries related to PD (QUEST-1-PD), administered to all patients
- Section 2: fears related to deep brain stimulation therapy (QUEST-2-DBS)
- Section 3: fears related to levodopa/carbidopa intestinal gel infusion (QUEST-3-LCIG)

Moreover, the following data were collected:

- Information about COVID-19
- Cognitive status, as per the Mini Mental State Examination (MMSE) score [12], obtained from the evaluation of the last 6 months by our clinical archives

### Statistical analysis

Descriptive statistics was used for continuous variables and frequency distribution for categorical data. Spearman correlation tests were performed to analyze correlations between HADS scores with age, disease duration, MMSE score, presence of caregivers, type of therapy, and QUEST-1-PD mean score, calculated by answers related to agreement or disagreement (10 questions; range score 1–5). Comparisons between patients and caregivers for sex, age, education, and HADS scores were analyzed by Mann-Whitney *U* test and chi-square test. Differences in the HADS scores and QUEST-1-PD at T0 between the three groups of patients were investigated using the non-parametric Kruskal-Wallis test. Post hoc analyses were run to correct for multiple comparisons. Wilcoxon test was used for the longitudinal comparisons of patients’ HADS scores between T0 and T1. All reported *p*-values are two-tailed. Statistical significance was set at *p* < 0.05. All data analyses were run using the Statistical Package for the Social Sciences (SPSS 26 for Windows, Chicago, IL).

## Results

**Fig. 1:**
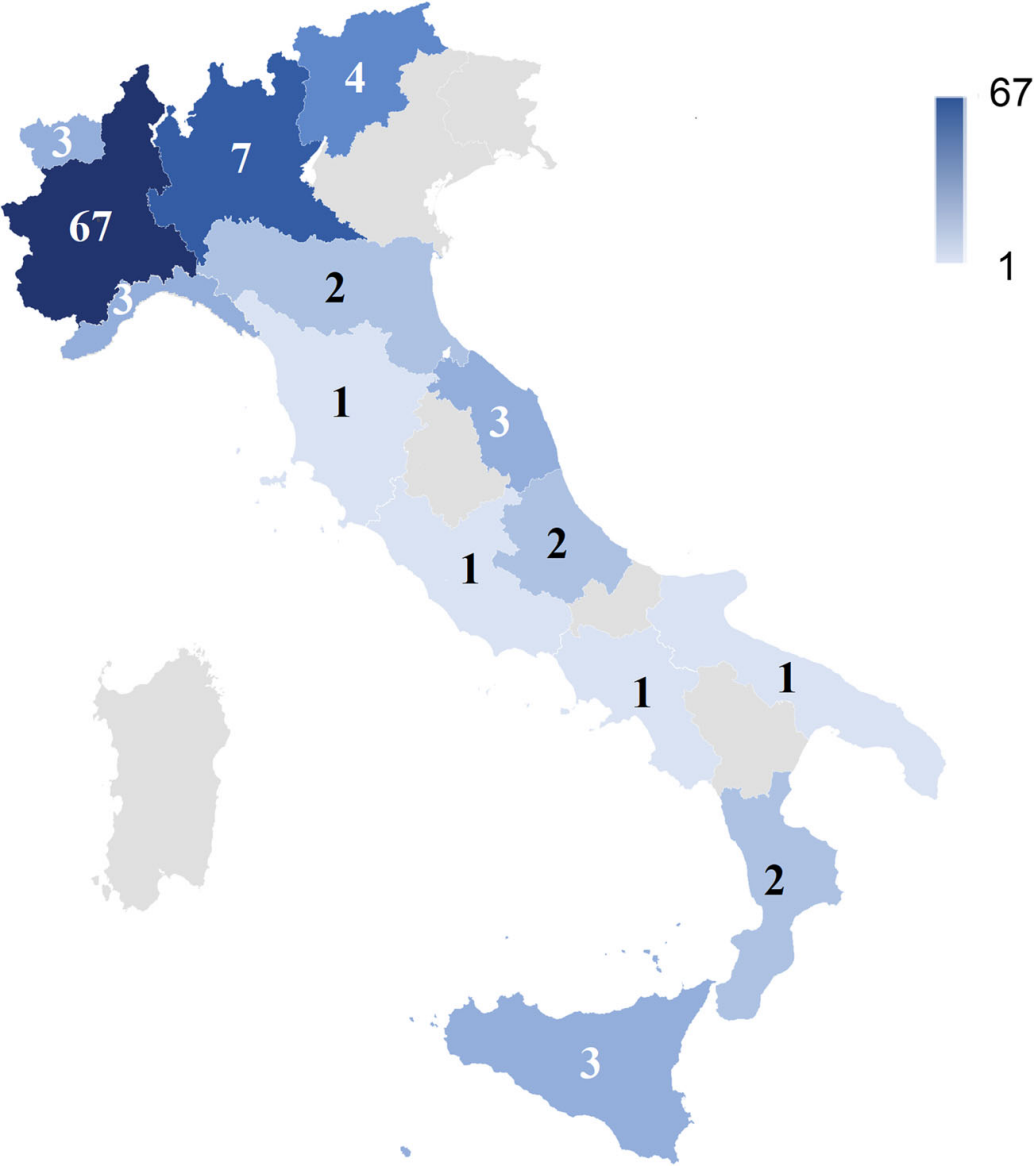
Distribution of our patients in the Italian Regions

We enrolled 100 APD patients and 60 caregivers; 54% of patients were treated with DBS, while 20% of them were on LCIG and 26% were on SMT; patients were from 14 Italian Regions (Fig. 1). The 11% of patients spent the lockdown period in isolation, while the 89% of them were overseen by at least one caregiver. Demographic and clinical characteristics of patients and caregivers are summarized in Tables 1 and 2.

**Table 1:** Patients’ demographic and clinical characteristics

	All patients (*n*= 100)	DBS group (*n*= 54)	LCIG group (*n*= 20)	SMT group (*n*= 26)	*p*-value
Age	62.4 ± 9.0 (38–78)	60.6 ± 9.0 (38–74)	69.6 ± 8* (47–78)	60.7 ± 7.1^#^ (43–72)	**0.000** *DBS vs LCIG: p=* ***0.000*** *DBS vs SMT: p= 1.000* *LCIG vs SMT: p=* ***0.000***
Sex (male/female)	60/40	31/23	13/7	16/10	n.a.
Education (years)	11.3 ± 4.2 (5–27)	12.1 ± 3.8 (5–27)	8.2 ± 4.7* (5–24)	12.2 ± 3.9# (8–18)	**0.000** *DBS vs LCIG: p=* ***0.000*** *DBS vs SMT:* *p= 1.000* *LCIG vs SMT: p=* ***0.001***
Employment	Employed *n*= 21 Not employed *n*= 14 Retired *n*= 65	Employed *n*=12 Not employed *n*= 10 Retired *n*= 32	Employed *n*= 2 Not employed *n*= 0 Retired *n*= 18	Employed *n*= 7 Not employed *n*= 4 Retired *n*= 15	n.a.
Disease duration (years)	13.4 ± 4.6 (6–31)	13.9 ± 4.9° (7–31)	14.7 ± 4.1 (8–23)	11.2 ± 3.7# (6–20)	**0.007** *DBS vs LCIG: p= 1.000* *DBS vs SMT:* *p=* ***0.028*** *LCIG vs SMT: p=* ***0.010***
CT duration (months)	n.a.	32.4 ± 21.1 (2–72)	35.7 ± 20.8 (4–74)	n.a.	n.a.
MMSE	28.5 ± 1.6 (24–30)	29.2 ± 1.1° (26–30)	27.4 ± 2.4* (24–30)	28.0 ± 1.3 (26–30)	**0.000** *DBS vs LCIG: p=* ***0.001*** *DBS vs SMT: p=* ***0.001*** *LCIG vs SMT: p= 1.000*
Symptoms observed from February 2020 to T0					n.a.
Flu symptoms Fever Cough Cold Respiratory difficulties Diarrhea Urinary burning Pneumonia (Further) smell reduction Taste reduction	*n*= 6 *n*= 7 *n*= 8 *n*=11 *n*=5 *n*=7 *n*=2 *n*=0 *n*=0 *n*=0	*n*= 4 *n*= 4 *n*= 7 *n*=8 *n*=3 *n*=5 *n*=1 *n*=0 *n*=0 *n*=0	*n*= 1 *n*= 1 *n*= 0 *n*=0 *n*=1 *n*=0 *n*=0 *n*=0 *n*=0 *n*=0	*n*= 1 *n*= 2 *n*=1 *n*=3 *n*=1 *n*=2 *n*=1 *n*=0 *n*=0 *n*=0	
Execution of nasopharyngeal swab (from February 2020 to T0)	*n*=0	*n*=0	*n*=0	*n*=0	n.a.
Diagnosis of Covid-19 (from February 2020 to T0)	*n*=0	*n*=0	*n*=0	*n*=0	n.a.
HADS (T0) HADS-A	6.7 ± 3.9 (0–16)	5.8 ± 3.7° (0–15)	8.1 ± 3.2* (3–15)	8.2 ± 4.4 (1–16)	**0.008** *DBS vs LCIG: p=* ***0.028*** *DBS vs SMT: p=* ***0.049*** *LCIG vs SMT: p= 1.000*
Normal (0–7) Mild (8–10) Moderate (11–15) Severe (≥16)	*n*= 61 *n*= 19 *n*= 19 *n*= 1	*n*= 39 *n*= 7 *n*= 8 *n*= 0	*n*= 9 *n*= 7 *n*= 4 *n*= 0	*n*= 13 *n*= 5 *n*= 7 *n*= 1	
HADS-D	6.5 ± 3.3 (0–17)	6.3 ± 3.6 (0–14)	7.3 ± 3.4 (1–17)	6.3 ± 2.5 (1–13)	0.494 No significant differences across samples
Normal (0–7) Mild (8–10) Moderate (11–15) Severe (≥16)	*n*= 65 *n*= 25 *n*= 9 *n*= 1	*n*= 33 *n*= 13 *n*= 8 *n*= 0	*n*= 12 *n*= 7 *n*= 0 *n*= 1	*n*= 20 *n*= 5 *n*= 1 *n*= 0	
HADS (T1) HADS-A	*n*=85 5.9 ± 3.7 (0–17)	*n*= 47 4.9 ± 3.6 (0–17)	*n*= 18 7.7 ± 2.8* (3–13)	*n*= 20 6.6 ± 3.8 (1–17)	**0.005** *DBS vs LCIG: p=* ***0.002*** *DBS vs SMT: p=* 0.065 *LCIG vs SMT: p=* 0.251
Normal (0–7) Mild (8–10) Moderate (11–15) Severe (≥16)	*n*= 59 *n*= 18 *n*= 6 *n*= 2	*n*= 38 *n*= 6 *n*= 2 *n*= 1	*n*= 9 *n*= 6 *n*= 3 *n*= 0	*n*= 12 *n*= 6 *n*= 1 *n*= 1	
HADS-D	6.6 ± 3.1 (1–14)	6.4 ± 2.8 (1–13)	7.9 ± 3.6 (3–14)	5.8 ± 2.9 (2–12)	0.159 No significant differences across samples
Normal (0–7) Mild (8–10) Moderate (11–15) Severe (≥16)	*n*= 56 *n*= 20 *n*= 9 *n*= 0	*n*= 30 *n*= 15 *n*= 2 *n*= 0	*n*= 11 *n*= 2 *n*= 5 *n*= 0	*n*= 15 *n*= 3 *n*= 2 *n*= 0	

All data are reported as mean ± standard deviation (range), with the exception of sex, employment, HADS anxiety and depression severity levels, symptoms observed from February 2020 to T0, execution of nasopharyngeal swab, and diagnosis of Covid-19. *n.a.*, not applicable; *MMSE*, Mini Mental State Examination; *T0*, data collected from April 2020 to May 2020, during the lockdown; *T1*, data collected after the end of the lockdown in Italy from June 2020 to August 2020; *HADS*, Hospital Anxiety and Depression Scale; *HADS-A*, HADS Anxiety subscale; *HADS-D*, HADS Depression subscale. Bold values mean statistically significant difference. Significant values adjusted by the Bonferroni correction for multiple tests are reported in italics

*Significant difference between DBS and LCIG

^#^Significant difference between LCIG and SMT

°Significant difference between DBS and SMT

**Table 2:** Caregivers’ demographic and clinical characteristics

Age	62.1 ± 9.2 (43–83)
Sex (male/female)	21/39
Education (years)	11 ± 3.9 (4–23)
Employment	Employed *n*= 20 Not employed *n*= 6 Retired *n*= 34
Symptoms observed from February 2020 to T0	
Flu symptoms Fever Cough Cold Respiratory difficulties Diarrhea Urinary burning Pneumonia Smell reduction Taste reduction	*n*=4 *n*=6 *n*=4 *n*=3 *n*=3 *n*=7 *n*=2 *n*=1 *n*=2 *n*=1
Execution of nasopharyngeal swab (fromFebruary 2020 to T0)	*n*=1
Diagnosis of Covid-19 (from February 2020 to T0)	*n*=1
HADS-A Normal (0–7) Mild (8–10) Moderate (11–15) Severe (≥16)	6.6 ± 4.6 (0–17) *n*= 36 *n*= 12 *n*= 11 *n*= 1
HADS-D Normal (0–7) Mild (8–10) Moderate (11–15) Severe (≥16)	5 ± 3.7 (1–17) *n*= 47 *n*= 8 *n*= 3 *n*= 2

All data are reported as mean ± standard deviation (range), with the exception of sex, employment, HADS anxiety and depression severity levels, symptoms observed from February 2020 to T0, execution of nasopharyngeal swab, and diagnosis of Covid-19. *HADS*, Hospital Anxiety and Depression Scale; *HADS-A*, HADS Anxiety subscale; *HADS-D*, HADS Depression subscale; *T0*, data collected from April 2020 to May 2020, during the lockdown

### Anxiety and depression assessment

At T0, HADS scores revealed that 39% of patients showed anxiety and 35% depression. At T1, the percentage was reduced for the anxiety (30.6%; *p*= 0.023) but not for the depression (34.1%; *p*= 0.807).

The QUEST-1-PD mean score showed a significant correlation with anxiety (*p*= 0.001) and a trend toward significance with depression (*p*= 0.077) at T0. The type of treatment was significantly correlated to the HADS-A score (*p*= 0.004), with LCIG and SMT patients showing the highest prevalence of anxiety.

At T0, we found no correlation between both anxiety and depression, and age (HADS-A score: *p*= 0.158; HADS-D score: *p*= 0.193), disease duration (HADS-A score: *p*= 0.987; HADS-D score: *p*= 0.559), number of caregivers (HADS-A score: *p*= 0.256; HADS-D score: *p*= 0.493), and MMSE score (HADS-A score: *p*= 0.821; HADS-D score: *p*= 0.057).

At T0, 40% of caregivers showed anxiety and 21.7% depression. A significant difference between patients and caregivers was found for HADS-D score (*p*= 0.001).

### Specific worries and distress related to COVID-19 and lockdown

**Fig. 2:**
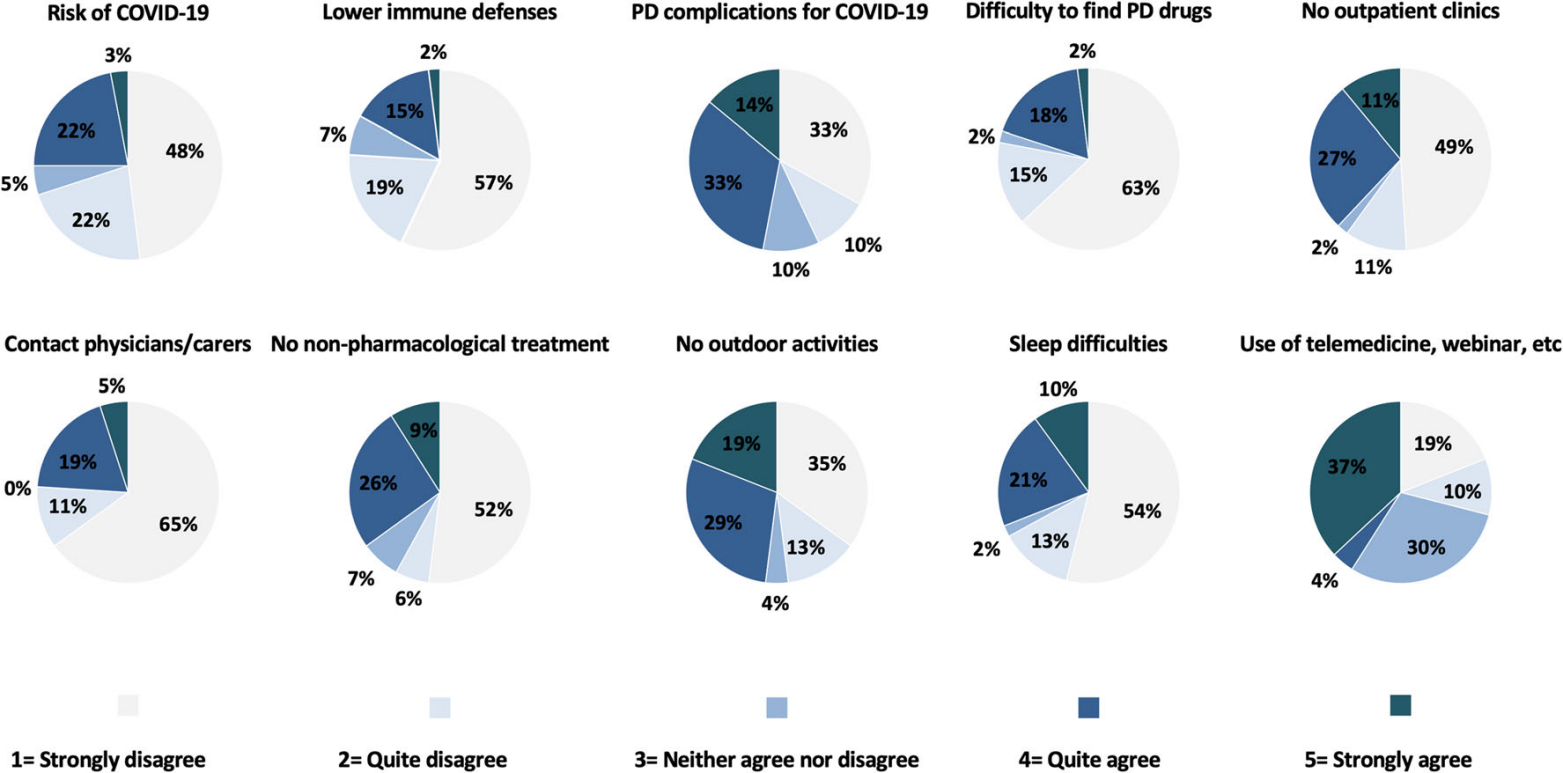
Patients’ answers to the QUEST-1-PD exploring distress and worries related to Parkinson disease during the COVID-19 outbreak reported as percentage

The 25% of patients were feared for a possible higher risk of infection, the 47% expressed worries about a possible worsening of their PD symptoms due to COVID-19 infection, the 20% about drug supplies, the 38% about limitation of access to hospital, and the 24% about the difficulty to consult with physicians or carers (Fig. 2). Moreover, the 35% of patients were worried about the interruption of non-pharmacological treatments, such as physiotherapy, psychological support, or cognitive stimulation, and the 48% about the possible worsening of symptoms consequent to the limitation of outdoor physical activity. The 39% of patients expressed a positive opinion about telemedicine, as well as webinars and toll-free numbers (Fig. 2).

### Impact of different therapies

Patients treated with SMT, LCIG, and DBS showed slight, albeit significant, differences in demographic and clinical features (Table 1).

At T0, the anxiety score was significantly higher in SMT and LCIG patients (HADS-A score: DBS vs LCIG *p*= 0.028; DBS vs SMT *p*= 0.050). At T1, the anxiety score was significantly higher in LCIG patients (*p*= 0.002) and maintained a trend toward statistical significance in SMT patients (*p*= 0.065). The three groups of patients did not differ for the presence of a caregiver during the COVID-19 outbreak and for the mean QUEST-1-PD score.

DBS patients reported specific worries about possible device-related issues: a percentage of 20.4% (*n*= 11) of them were worried about the difficulty to consult the neurologist for a modification of the stimulation parameters, a percentage of 24.1% (*n*= 13) about the stimulator charge and the possible difficulty for replacement, and a percentage of 40.7% (*n*= 22) about the interruption of regular follow-up visits. In addition, 35.2% (*n*= 19) of patients were worried that their caregiver contracted COVID-19.

LCIG patients reported worries about infection of the stoma (35%, *n*= 7) or a probe block or dislocation (30%, *n*= 6). In addition, a large proportion of them (40%, *n*= 8) were worried of a possible block of intestinal infusion of levodopa, or that their caregiver got affected by COVID-19 (30%, *n*=6).

## Discussion

We evaluated the psychological impact of the COVID-19 pandemic on APD patients and their caregivers, analyzing specific worries in a large sample of patients who were stratified for treatment strategies. Anxiety and depression occurred in more than 30% of patients during the lockdown interval, with improvement of anxiety in the months that followed the end of lockdown. This confirms previous data on the negative effect of the COVID-19 outbreak on PD [4–6], and extend them to patients at advanced disease stages.

Our study highlighted a differential psychological impact in relation to the treatment of APD patients. Anxiety correlated with specific distress/worries, investigated by an ad hoc questionnaire. Most impacting distress factors on patients included their perceived higher risk to develop COVID-19 with a worse outcome, difficulty to have hospital access, and interruption of non-pharmacological treatments. Patients treated with DAT reported also specific worries related to their device dysfunction and the risk for their caregiver to suffer from COVID-19. In addition, significant anxiety and depression were observed also in a relevant proportion of caregivers (40% and 21.7%, respectively).

We argue that the absence of reliable data on the risk for developing COVID-19 in patients with PD [7] and poor information induced feelings of doubts and uncertainty, which increased the level of anxiety; indeed, the need of a correct information during the outbreak has been claimed by a recent study [8]. Worries and distress about PD symptoms could also arise from the restrictions of the lockdown. Physical activity and non-pharmacological interventions are relevant tools for managing PD symptoms [13, 14] and the interruption or limitation of these activities has remarkably contributed to increase negative feelings in our patients. Moreover, APD patients need frequent outpatient visits; in fact, one of the major concerns reported in our interview was related to the limitation of outpatient clinic access and possible difficulties to consult with physicians. The limitation of access to healthcare services can be, at least partially, mitigated by telemedicine [8, 15], and this issue was appreciated or advocated by almost half of the patients, underlying the relevance of this different modality of assistance.

Interestingly, our data showed a correlation between the type of treatment and anxiety, while no correlation was observed with depression. This indicates that anxiety was likely related to the strain for PD symptoms management during the outbreak. Indeed, patients treated with DAT expressed fears about the possibility to obtain adequate and rapid healthcare assistance in case of device dysfunction. Another frequent concern was the risk for the caregiver to develop COVID-19. The caregiver is indeed regarded by patients as an essential resource for both symptom management and psychological support [16]. Noteworthy, anxiety levels were lower in DBS-treated patients than in LCIG- and SMT-treated patients, probably for the relatively independent management of the DBS device.

Our data confirm the findings of other studies that investigated the psychological impact of COVID-19 on PD patients, highlighting significant emotional sequelae of the pandemic. In particular, depression was previously reported to occur in 21% of patients, and anxiety in a percentage ranging from 21 to 81.7% of patients [17] and in 57.9% of caregivers [5]. In addition, increased distress was reported in 43.8% of patients and 53.1% of caregivers [4].

Our data assessed the psychological consequences of COVID-19 pandemic in a specific group of PD patients, i.e., those with APD, highlighting different emotional and behavioral responses in relation to the type of treatment. This indicates that specific needs should be taken into account for APD patients. Previous studies assessing the impact of COVID-19 pandemic in patients with PD suggested that the presence of neuropsychiatric symptoms before the pandemic, as well as cognitive dysfunction, could predict an increased psychological distress [18]. This result further supports our findings on a cohort of APD patients, especially those treated with DAT, and suggests a strict monitoring for neuropsychiatric issues. In this context, telemedicine could be a good option, with a high level of acceptance by patients according to our questionnaire and results of previous studies [19].

The main limitation of our study is the lack of baseline evaluations of anxiety and depression before the COVID-19 outbreak. However, the comparison between two time-points (T0 and T1) has likely detected a remarkable part of the effects of the pandemic. Another aspect to be taken into account is that our cohort focused mostly on APD patients treated with DAT. Encompassing patients with different therapeutic approaches may be considered a strength of the study; however, the presence of only 26% of APD patients treated with SMT should be considered in the generalization of our findings.

In conclusion, we identified multiple reactions and emotional states in different groups of APD patients, underlying the necessity to strictly monitor the psychological impact of the pandemic in such frail a population. Finally, specific information strategies and education campaigns are necessary to reduce distress and anxiety for both patients and caregivers.

## **Supplementary information**

Questionnaire administered to our patients to investigate specific worries related to the pandemic.

ESM 1 (DOCX 25 kb)

## Data Availability

The datasets generated during and/or analyzed during the current study are available from the corresponding author on reasonable request.
