# Supplementary material for: Anxiety, depression, and worries in advanced Parkinson disease during COVID-19 pandemic
Source: Neurol Sci. 2021 May 4;43(1):341–8. doi: 10.1007/s10072-021-05286-z (PMC8096160; doi:10.1007/s10072-021-05286-z)
Supplement: Supplementary file 1 — (DOCX 25 kb) [file 10072_2021_5286_MOESM1_ESM.docx]

**QUESTIONNAIRE**

**SECTION 1: DISTRESS AND WORRIES RELATED TO PARKINSON DISEASE (QUEST-1-PD)**

|  | ***Strongly disagree*** | ***Quite disagree*** | ***Neither agree nor disagree*** | ***Quite***  ***agree*** | ***Strongly agree*** |
| --- | --- | --- | --- | --- | --- |
| 1- I am afraid that having Parkinson disease could expose me to a greater risk to be infected by COVID-19 | 1 | 2 | 3 | 4 | 5 |
| 2- I am afraid that pharmacological therapy for Parkinson disease could lower my immune defenses | 1 | 2 | 3 | 4 | 5 |
| 3- I am afraid of complications due to Parkinson disease in case of COVID-19 | 1 | 2 | 3 | 4 | 5 |
| 4- I am worried about the possible difficulty to find drugs | 1 | 2 | 3 | 4 | 5 |
| 5- I am worried for the interruption of outpatient clinics | 1 | 2 | 3 | 4 | 5 |
| 6- I am afraid about the difficulties to contact physicians or carers in case of need | 1 | 2 | 3 | 4 | 5 |
| 7- I am afraid for non-pharmacological treatment interruption (i.e. physiotherapy, psychological support, cognitive stimulation) | 1 | 2 | 3 | 4 | 5 |
| 8- I am afraid for parkinsonian symptoms worsening due to prohibition to do outdoor physical activity | 1 | 2 | 3 | 4 | 5 |
| 9- I have more difficulty in falling asleep | 1 | 2 | 3 | 4 | 5 |
| 10- I found useful to make use of telemedicine, consult webinars, or call a dedicated toll-free number | 5 | 4 | 3 | 2 | 1 |

**SECTION 2: FEARS RELATED TO DEEP BRAIN STIMULATION (QUEST-2-DBS)**

|  | ***Strongly disagree*** | ***Quite disagree*** | ***Neither agree nor disagree*** | ***Quite***  ***agree*** | ***Strongly agree*** |
| --- | --- | --- | --- | --- | --- |
| 11- I am worried about the difficulty to contact the neurologist to adjust the stimulation parameters if necessary | 1 | 2 | 3 | 4 | 5 |
| 12- I am afraid that the stimulation battery could exaust and that their replacement may take a long time | 1 | 2 | 3 | 4 | 5 |
| 13- I am worried about the interruption of regular follow-up visits to verify the current stimulation parameters | 1 | 2 | 3 | 4 | 5 |
| 14- I have noticed the onset of these thoughts and emotions since December 2019  YES NO |  | | | | |
| 15- I have noticed the increase of these thoughts and emotions since December 2019  YES NO |  | | | | |
| 16- I am afraid that my caregiver may be infected by COVID-19 and no longer support me | 1 | 2 | 3 | 4 | 5 |

**SECTION 3: FEARS RELATED TO LEVODOPA/CARBIDOPA INTESTINAL GEL INFUSION (QUEST-3-LCIG)**

|  | ***Strongly disagree*** | ***Quite disagree*** | ***Neither agree nor disagree*** | ***Quite***  ***agree*** | ***Strongly agree*** |
| --- | --- | --- | --- | --- | --- |
| 17- I am afraid about an infection in the stoma and the difficulty to obtain adequate health care | 1 | 2 | 3 | 4 | 5 |
| 18- I am afraid that intestinal gel infusion may stop | 1 | 2 | 3 | 4 | 5 |
| 19- I fear that the probe will come off, become blocked or dislocated and that it is difficult to obtain adequate health care | 1 | 2 | 3 | 4 | 5 |
| 20- I have noticed the onset of these thoughts and emotions since December 2019  YES NO |  | | | | |
| 21- I have noticed the increase of these thoughts and emotions since December 2019  YES NO |  | | | | |
| 22- I am afraid that my caregiver may be infected by COVID-19 and no longer support me or help me with the device management | 1 | 2 | 3 | 4 | 5 |
